# Endothelial Cells Differentiated from Human Induced Pluripotent Stem Cells Form Aligned Network Structures in Engineered Neural Tissue

**DOI:** 10.3390/jfb16110425

**Published:** 2025-11-20

**Authors:** Poppy O. Smith, Parmjit Jat, James B. Phillips

**Affiliations:** 1 UCL Centre for Nerve Engineering, Department of Pharmacology, UCL School of Pharmacy, University College London, London WC1N 1AX, UK; 2 MRC Prion Unit at UCL and Institute of Prion Diseases, University College London, London W1W 7FF, UK; p.jat@prion.ucl.ac.uk

**Keywords:** nerve tissue engineering, endothelial cells, human induced pluripotent stem cells (hiPSCs), engineered neural tissue, gel aspiration-ejection, collagen hydrogel, aligned endothelial structures

## Abstract

Background/Objectives: Endothelial cells play a key role in peripheral nerve regeneration, forming aligned vasculature which bridges the gap in the injured nerve tissue and guides the regrowing tissue. This work aimed to mimic key features of this aligned vasculature by differentiating endothelial cells from human induced pluripotent stem cells (hiPSCs) and incorporating them into engineered neural tissue (EngNT). Methods: hiPSCs were differentiated into endothelial cells with the temporal addition of growth factors and biomolecules. These hiPSC-derived endothelial cells (hiPSC-ECs) were incorporated into EngNT fabricated from collagen hydrogels using the gel aspiration-ejection (GAE) technique and maintained in vitro to allow endothelial network formation. Results: At the mRNA and protein level, pluripotency marker expression decreased and endothelial cell marker expression increased over the course of hiPSC differentiation to endothelial cells. The derived endothelial cells expressed CD31, CD144, ENG, VEGFR2, and VWF, and formed network structures in the matrix tubulogenesis assay. hiPSC-ECs incorporated into EngNT were viable and aligned. They formed highly aligned tube-like structures containing lumens after four days in culture and the EngNT constructs supported neurite growth in vitro when co-cultured with rat dorsal root ganglion (DRG) neurons. Conclusions: This work rapidly generated engineered nerve tissue containing highly aligned endothelial tube-like structures, resembling key features of the early nerve regeneration bridge. Therefore, this 3D engineered tissue provides a platform to study the effects of endothelial cell structures in nerve repair treatment and translational development.

## 1. Introduction

Peripheral nerve injuries impact millions of people every year, resulting in loss of function and sensation with potential long-term neuropathic pain and lifelong disability [1]. Fortunately, the peripheral nervous system has an intrinsic ability to regenerate following injury when provided with a supportive environment where a connection is formed between the ends of the damaged nerve [2]. Endothelial cells play multiple support roles in nerve regeneration and repair. As in all tissues, endothelial cells are required to form the blood vessels necessary to vascularise nerve tissue. Vascularisation is required for long-term tissue survival and provides a transport system for the supply of oxygen and nutrients, and the removal of waste [3]. Pre-vascularised engineered nerve tissues have been shown to integrate with the host vasculature and provide a conducive environment for axon re-growth and functional recovery in long sciatic nerve defects in rabbits and rodents [4,5,6]. Beyond their vascular function, endothelial cells secrete various factors beneficial to regeneration [7], such as brain-derived neurotrophic factor (BDNF) which when secreted by human umbilical vein endothelial cells (HUVECs) enhanced chicken and rat dorsal root ganglion neuron outgrowth in vitro [8]. Exosomes secreted from HUVECs have also been shown to promote the repair phenotype in cultures of RSC96 Schwann cells and improve functional recovery and remyelination in a rat sciatic nerve crush model when injected following injury [9].

Following a nerve transection injury in rodents, endothelial cells in both nerve stumps undergo angiogenesis and can migrate through the tissue bridge that forms across the nerve gap, forming multiple thin blood vessels which link the two nerve stumps. This polarised structure guides the subsequent migration of Schwann cells and provides a path for axonal regeneration [10]. Disruption of organised vascular growth through genetic manipulation of Plexin-D1 in mice resulted in the abnormal regrowth of nerve fibres across the nerve bridge, delaying regeneration [11]. The maximal distance between nerve stumps that can support spontaneous formation of this endothelial cell bridge has not been determined, but the length is typically a few millimetres in animal models. The contribution of organised vascular structures to the support and guidance of nerve regeneration across small gaps following transection raises the possibility that mimicking such features could be a useful tool in nerve tissue engineering for bridging larger gaps in humans, which are generally treated using a nerve autograft [2]. Our previous work indicated that collagen gels containing aligned structures made from HUVECs supported nerve regeneration in a rat nerve gap model to a greater extent than equivalent materials containing Schwann cells [5]. To progress nerve tissue engineering research based on this concept, the present study aimed to develop EngNT containing organised and stable tube-like structures formed from endothelial cells suitable for translational development as an advanced therapy medicinal product (ATMP).

Human endothelial cells can be isolated from patients, with endogenous sources of mature and progenitor endothelial cells in adults found in the peripheral blood, blood vessels, bone marrow, and adipose tissue [12,13,14]. While autologous cells would reduce the likelihood of graft rejection, the number of autologous cells suitable for isolation is limited and endothelial cells are subject to inter-organ and inter-patient variability [15,16]. Furthermore, in nerve tissue engineering to treat trauma, rapid repair is desirable, increasing the possibility of a full recovery [17]. Therefore, allogeneic human induced pluripotent stem cell (hiPSC) derived cells, which have recently gained much attention in Parkinson’s disease clinical trials [18], could be suitable for an ‘off-the-shelf’ application, overcoming the time-consuming harvesting and processing of patient-derived autologous cells [19,20,21]. Endothelial cells differentiated from hiPSCs [22,23] could potentially be used as part of an allogenic engineered tissue approach. Issues around immunogenicity and graft rejection could be addressed through the use of human leukocyte antigen (HLA) haplotype matching, HLA cloaking, or immunosuppression, and risks of tumorigenicity mitigated by stringent purification and the use of inducible suicide genes [20,24]. In this study, endothelial cells were differentiated from hiPSCs and characterised at the molecular, protein, and functional level. Subsequently, collagen hydrogel-based engineered neural tissue (EngNT) containing the derived endothelial cells (EngNT-ECs) was produced and the formation of stable aligned tube-like structures within the constructs was investigated. The potential of the EngNT-EC constructs to support neuronal regeneration was then assessed in vitro. This work advanced engineered nerve tissue by incorporating endothelial luminal structures that mimic key features of the vascular structures seen early in the natural nerve regeneration process. This provides a platform suitable for investigating the role of aligned vascular structures in peripheral nerve injury treatment and translational development.

## 2. Materials and Methods

### 2.1. Cell Culture and Differentiation

Human CTX-iPSC-1.4 (hiPSCs) cells (ReNeuron Ltd., Pencoed, UK) were cultured in mTeSR™-Plus Basal Growth Medium supplemented with mTeSR™-Plus Supplement (Stem Cell Technologies, Cambridge, UK) on vitronectin (ThermoFisher, Paisley, UK) coated plates. hiPSCs were regularly passaged using 0.5 mM UltraPure EDTA™ (Life Technologies, Paisley, UK) and replated as small cell clumps at dilutions of 1:4 to 1:6, with media changes every 2–3 days. hiPSCs were used from Passage 8 to Passage 18. All cell cultures and engineered tissues were maintained in a humidified incubator at 37 °C with 5% CO_2_/95% air.

**Figure 1:**
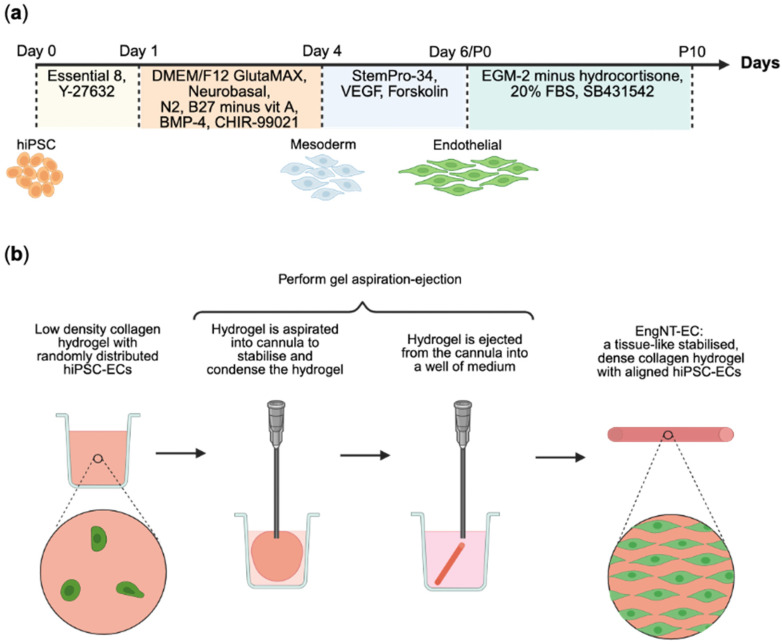
Production of engineered neural tissue containing human induced pluripotent stem cell-derived endothelial cells (EngNT-ECs). (**a**) Schematic illustration of the differentiation of human induced pluripotent stem cells (hiPSCs) into endothelial cells. (**b**) Schematic summary of the formation of EngNT from a low-density collagen hydrogel containing randomly aligned cells to a tissue-like, stabilised, compacted collagen hydrogel with cells aligned along the construct longitudinal axis. Created in BioRender 2024.

hiPSC to endothelial cell differentiation was performed largely following the protocol developed by [25]. Briefly, hiPSCs were dissociated with 0.5 mM UltraPure EDTA™ and plated on Geltrex™ (ThermoFisher, Paisley, UK) coated plates at a density of 26,500 cells/cm^2^ in Essential 8™ Medium (Gibco, Paisley, UK) supplemented with 10 µM Rho-associated protein kinase (ROCK) inhibitor, Y-27632 (Miltenyi Biotec, Woking, UK). After 24 h (Day 1), the medium was changed to a 1:1 mix of DMEM/F12 GlutaMAX (Gibco, Paisley, UK) and Neurobasal (Gibco, Paisley, UK) with 1X N2 and 1X B27 minus vitamin A supplements, plus 25 ng/mL human recombinant bone morphogenetic-protein 4 (BMP4) (Bio-Techne, Abingdon, UK) and glycogen synthase kinase-3 (GSK-3) inhibitor, CHIR-99021 (Bio-Techne, Abingdon, UK). After 3 days (Day 4), the medium was changed to StemPro™-34 (Life Technologies, Paisley, UK) supplemented with 200 ng/mL human recombinant vascular endothelial growth factor-165 protein (VEGF-165) (PeproTech, London, UK) and 2 µM forskolin (Cambridge Bioscience, Cambridge, UK). This medium was renewed 24 h later (Day 5). On Day 6, cells were dissociated with Accutase (Sigma, Dorset, UK) and cultured at 40,000 cells/cm^2^ on Geltrex™-coated plates in Endothelial Growth Medium-2 (EGM-2) Endothelial Medium Bulletkit (Lonza, Slough, UK) without hydrocortisone, and further supplemented with 20% foetal bovine serum (FBS) (Sigma, Dorset, UK), 10 µM SB431542 (Bio-Techne), and 100 U/mL penicillin and 100 µg/mL streptomycin (Pen-Strep) (Sigma, Dorset, UK) (Figure 1a). Cells were passaged every 3–4 days at dilutions of 1:3 or cryopreserved in 70% *v*/*v* medium, 20% *v*/*v* FBS, and 10% *v*/*v* DMSO (Sigma, Dorset, UK).

### 2.2. Tubulogenesis Assay

To assess the ability of the hiPSC-derived endothelial cells to form tube-like structures when seeded atop an extracellular matrix hydrogel, a key function of endothelial cells, tubulogenesis assays were conducted as previously described [26] with 10,000 CD31 and CD144 double-positive cells per well of a 96-well plate seeded on Corning™ Matrigel™ GFR Membrane Matrix (Thermo Scientific, Paisley, UK). Tubulogenesis assays were monitored on an Incucyte^®^ S3 (Sartorius, Epsom, UK), taking phase contrast images every hour.

### 2.3. Tissue Engineering

Cellular hydrogels were formed using Type I rat tail collagen as described previously [27] with a cell density of 2.0 × 10^6^/mL. In summary, for 1 mL collagen hydrogel, 800 µL 2 mg/mL Rat Tail Type I Collagen in 0.6% acetic acid (FirstLink UK Ltd., Birmingham, UK) was added to 100 µL 10X Minimum Essential Medium (Sigma, Dorset, UK) and neutralised using sodium hydroxide (Fisher Scientific, Loughborough, UK). Upon neutralisation, 2.0 × 10^6^ cells were added in 100 µL high glucose Dulbecco’s Modified Eagle Medium (DMEM) (Sigma, Dorset, UK) and 1 mL of the solution was cast into a 48-well plate and set for 30 min in a humidified incubator at 37 °C. Cellular hydrogels were simultaneously stabilised and aligned using automated gel aspiration-ejection (GAE) [28], by drawing the hydrogel into a 16 gauge (16G) (internal diameter 1.19 mm) blunt-ended cannula (VWR International Ltd., Lutterworth, UK) under negative pressure with an aspiration rate of 35 µL/s (Figure 1b). The resulting stabilised hydrogel was ejected at 50 µL/s to give the EngNT constructs containing the hiPSC-derived endothelial cells (EngNT-EC), which were then cultured in the supplemented EGM-2 with daily medium changes.

### 2.4. Viability Assays

After 24 h in culture, EngNT-EC viability was quantified using the Lactate Dehydrogenase (LDH) Assay Kit (Cytotoxicity) (Abcam, Cambridge, UK) or ReadyProbes™ Cell Viability Imaging Kit Blue/Red (Invitrogen, Paisley, UK) as per the manufacturer’s protocols. LDH assay percentage death was calculated using dead controls which had been incubated with 1% *v*/*v* Triton™ X-100 (Sigma, Dorset, UK) in Dulbecco’s phosphate-buffered saline (D-PBS) (Gibco, Paisley, UK) for 2 h before performing the assay.

### 2.5. Reverse Transcription–Quantitative Polymerase Chain Reaction (RT-qPCR)

Monolayer cells and EngNT-EC constructs were lysed in RLT+ RNeasy lysis buffer, with the additional step of disruption in Qiagen TissueLyser II for the EngNT-EC constructs. Total RNA was extracted using the RNeasy Plus Mini Kit (Qiagen, Manchester, UK) according to the manufacturer’s protocol. cDNA was prepared by reverse transcription using GoScript Reverse Transcriptase Kit (Promega, Chilworth, UK) and Applied Biosystems SimpliAmp Thermal Cycler (Applied Biosystems, Paisley, UK). RT-qPCR samples were run in duplicate using Power SYBR™ Green PCR Master Mix (ThermoFisher, Paisley, UK) with an Applied Biosystems QuantStudio3 instrument (Applied Biosystems, Paisley, UK) and analysed with QuantStudio Design and Analysis Software v1.5.1 (Applied Biosystems, Paisley, UK). The amplification products were analysed by performing a melting curve at the end of the PCR. Data were normalised to the expression of three reference genes: *HPRT1*, *RPLP0*, and *RPS18* [29,30,31]. Expression data represented as fold change versus the experimental control with the expression of the control group set to 1. Supplementary Table S1 lists the RT-qPCR primer sequences.

### 2.6. Neurite Extension Assay

Adult female Sprague Dawley rats (250–300 g) were culled humanely by exposure to an increasing concentration of carbon dioxide gas, followed by cervical dislocation, in accordance with the UK Animals (Scientific Procedures) Act 1986 (Appropriate Methods of Humane Killing) Order 1996 approved by UCL Animal Welfare and Ethics Review Board (AWERB). DRGs were dissected from the rat spines, with 23–30 DRGs collected per animal. Following the dissection of the DRGs, ganglia were incubated in 0.125% *w*/*v* collagenase type IV (Sigma, Dorset, UK) in high glucose DMEM for 90 min at 37 °C. Trituration dissociated the DRGs, the product of which was washed thrice in 10 mL high glucose DMEM supplemented with 10% *v*/*v* FBS and 100 U/mL penicillin and 100 µg/mL streptomycin. The dissociated cell suspension was incubated with 0.01 mM cytosine arabinoside (Ara-C) (Sigma, Dorset, UK) in fully supplemented high glucose DMEM on poly-D-lysine (PDL) (Sigma, Dorset, UK) coated flasks for 48 h. After incubation, cells were collected with 0.25% trypsin-EDTA (Gibco, Paisley, UK), centrifuged at 400× *g* for 5 min, and resuspended in supplemented high glucose DMEM and counted, yielding approximately 13,000 cells per DRG. This cell suspension was then seeded onto longitudinally halved EngNT-EC constructs, 2 µL each containing ~10,000 cells. After 15 min incubation to encourage DRG-derived neuron adherence, constructs were incubated in 3 mL basal EGM-2 for 72 h. Samples were fixed in 4% PFA overnight at 4 °C before immunofluorescence staining.

### 2.7. Immunofluorescence Staining

After fixation, monolayer cells were permeabilised and blocked in 0.5% *v*/*v* Triton™ X-100 in phosphate-buffered saline (PBS) (Sigma, Dorset, UK) containing 5% horse or goat serum (Abcam, Cambridge, UK) for 30 min at room temperature. Cells were stained overnight at 4 °C with primary antibody (see Table 1) in staining buffer containing 0.5% Triton™ X-100 and 1% serum. Cells were washed five times in 0.5% Triton X-100 before incubation with secondary antibody (see Table 1) and Hoechst 33342 (ThermoFisher, Paisley, UK) at 1:1000 in staining buffer for 1 h at room temperature. Cells were washed thrice with 0.5% Triton™ X-100 and twice with PBS before imaging. The same was performed for EngNT construct staining with a longer permeabilisation of 1 h, using Rhodamine 110 Phalloidin (Biotium, Cambridge, UK) at 1:40 dilution or Alexa Fluor^®^ 488 Anti-beta III tubulin antibody (Abcam, Cambridge, UK).

EngNT-EC constructs used for cryosectioning were then cryoprotected in 15% and then 30% *w*/*v* sucrose (Sigma, Dorset, UK) in PBS for 24 h at each concentration, then embedded in Neg-50™ Frozen Section Medium (ThermoFisher, Paisley, UK) and frozen in liquid nitrogen-cooled isopentane (Sigma, Dorset, UK). EngNT constructs were cut into 10 µm thick transverse sections using a HM525 Cryostat (ThermoFisher, Paisley, UK). Sections were mounted using VECTASHIELD^®^ Vibrance™ Antifade Mounting Medium (VectorLabs, London UK) on Epredia™ SuperFrost Plus™ Adhesion slides (ThermoFisher, Paisley, UK) for imaging.

### 2.8. Microscopy and Image Analysis

Phase contrast images were captured at 10× magnification using a Sartorius Incucyte^®^ S3 with Adherent Cell-by-Cell Module. Tubulogenesis assay images were analysed using the Angiogenesis Analyzer plugin in ImageJ 1.53a [32]. Fluorescence images were captured using a high-content screening confocal spinning disc microscope, Opera Phenix (Perkin Elmer, High Wycombe, UK), at 20× magnification at predetermined positions. Columbus Image Analysis Software v2.9.1 (Perkin Elmer, High Wycome, UK) was used to analyse positive cell numbers.

A Zeiss LSM 710 confocal microscope was used to capture z-stacks for EngNT-EC construct viability (20 µm z-depth), alignment (20 µm z-depth), tube-like structure formation (40 µm z-depth), and neurite extension (20–56 µm z-depth) at predetermined positions. Viability and cell and neurite alignment were analysed using 3D image analysis software, Volocity™ v6.5.1 (Perkin Elmer, High Wycombe, UK). Maximum intensity projections of tube-like structures were analysed using the Angiogenesis Analyzer plugin in ImageJ. Neurite length was analysed from maximum intensity projections in ImageJ.

Cross-sections of EngNT-EC constructs were captured using a Zeiss AxioLab A1 upright fluorescence microscope (Carl Zeiss Microscopy Ltd., Cambridge, UK) at 20× magnification at predetermined positions. Lumens, defined as a complete ring of staining with no staining in the centre, with a diameter between 10 µm and 50 µm were counted using ImageJ.

### 2.9. Statistical Analysis

All data, other than box and whisker plots, are presented as mean ± SEM. A Shapiro–Wilk test was used to determine normality of data distribution. Comparisons between two groups were performed using a two-tailed unpaired *t*-test or a Mann–Whitney test, depending on normality. Comparison between three or more groups was performed using a one-way ANOVA with a Dunnett’s or Tukey’s post hoc test, or a Kruskal–Wallis with a Dunn’s post hoc test, depending on normality. For all statistical analyses * *p* < 0.05, ** *p* < 0.01, *** *p* < 0.001, and **** *p* < 0.0001.

## 3. Results

The present study aimed to provide a readily available source of human endothelial cells for nerve tissue engineering from hiPSCs, which could be used to form aligned vascular-like structures within a stabilised collagen matrix, thereby mimicking the early vascular structures seen in native regenerating peripheral nerve tissue.

### 3.1. Endothelial Cells Were Successfully Differentiated from hiPSCs

**Figure 2:**
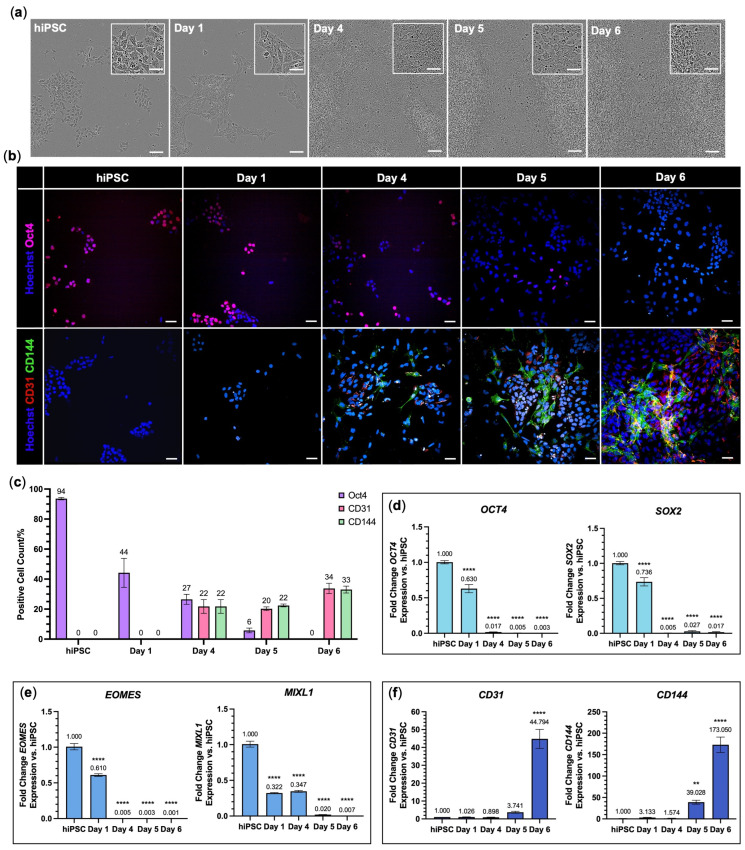
Differentiation of hiPSCs to endothelial cells. (**a**) Representative phase contrast images at various time points throughout differentiation. Scale bar 100 µm, with 50 µm for inset. (**b**) Representative immunofluorescence images show the proportion of cells positive for pluripotency marker Oct4 (magenta) and the proportion of cells positive for endothelial cell markers CD31 (red) and CD144 (green) throughout differentiation. All cells stained with Hoechst 33342 (blue). Scale bar 50 µm. (**c**) Columbus Image Analysis Software quantification of immunofluorescence by percentage positive cells for Oct4 (purple), CD31 (pink), and CD144 (green) at various time points throughout differentiation. (**d**–**f**) Fold change in mRNA expression of (**d**) pluripotency markers OCT4 and SOX2, (**e**) mesoderm markers EOMES and MIXL1, and (**f**) endothelial cell markers CD31 and CD144 relative to hiPSCs. Data are mean ± SEM with one-way ANOVA with Dunnett’s comparison test; ** *p* < 0.01, **** *p* < 0.0001. N = 3 independent differentiation inductions with n = 3 technical replicates.

Differentiation of hiPSCs to endothelial cells was conducted following the Hildebranst et al. protocol [25], a modified version of the well-cited Patsch et al. protocol [33], with the additional change in culturing the resulting hiPSC-derived cells on Geltrex™-coated tissue culture plastic, instead of gelatin. hiPSCs began as circular cells grouped in colonies. From Day 1, the transition of hiPSCs to mesoderm caused a flattening of the cell shape and changes in cell morphology from circular, compact colonies to elongated polygonal cells (Figure 2a). This was accompanied by a decrease in the pluripotency marker, Oct4, and an increase in expression of endothelial cell markers, CD31 and CD144, yielding 33.8 ± 2.0% and 33.0 ± 1.3% of cells positive for CD31 and CD144 on Day 6, respectively (Figure 2b,c). PCR was used to characterise the differentiation, showing significant downregulation of *OCT4* and *SOX2* (*p* < 0.0001 range; Figure 2d); expression of mesoderm markers *EOMES* and *MIXL1* decreased upon endothelial cell specification from Day 4 (*p* < 0.0001 range; Figure 2e); and *CD31* and *CD144* expression was upregulated from Day 5 (*p* = 0.0001–0.0096; Figure 2f).

**Figure 3:**
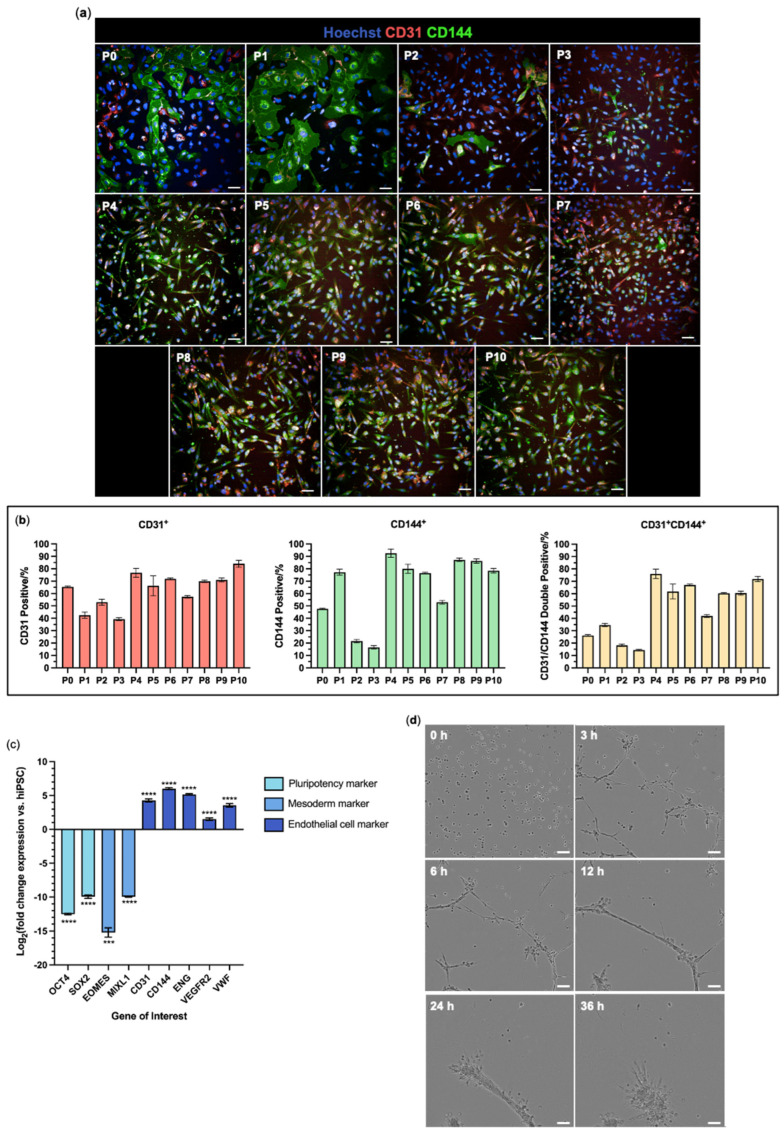
Expansion and characterisation of hiPSC-derived endothelial cells. (**a**) Differentiated hiPSCs were expanded in culture and immunoreactivity for endothelial cell markers CD31 (red) and CD144 (green) was assessed across Passage 0 (P0) to Passage 10 (P10), spanning 35 days in culture. All cells were stained with Hoechst 33342 (blue). Scale bar 50 µm. (**b**) Columbus Image Analysis quantification by percentage positive CD31^+^ (red), positive CD144^+^ (green), and double positive CD31^+^CD144^+^ (yellow). (**c**) Log2-fold change mRNA expression of pluripotency markers *OCT4* and *SOX2*, mesoderm markers *EOMES* and *MIXL1*, and endothelial cell markers *CD31, CD144, ENG, VEGFR2,* and *VWF* in hiPSC-ECs P4 relative to hiPSCs. (**d**) Representative phase contrast images of hiPSC-ECs at Passage 4 during the matrix gel tubulogenesis assay. The imaging frame was consistent over time. See Video S1 for the corresponding time-lapse video. Scale bar 100 µm. Data are mean ± SEM with one-way ANOVA with Tukey’s or Dunnett’s comparison test; *** *p* < 0.001, **** *p* < 0.0001. See Table S2 for full statistics on (**b**). N = 3 independent differentiation inductions with n = 3 technical replicates.

Following Day 6, the hiPSC-derived cells were cultured for 10 passages under vascular endothelial cell-favouring conditions. At each passage, the proportion of cells positive for CD31 and CD144 immunoreactivity was assessed (Figure 3). Following initial fluctuations during the first three passages, 76.7 ± 3.6% of cells were positive for CD31 at P4 and there were no significant differences in the percentage of CD31^+^ cells between Passages 4 and 10, except for Passage 7 (Figure 3b) (see Table S2 for statistical analysis data). Passage 4 also showed the highest proportion of CD144^+^ cells (92.6 ± 3.4%) and double-positive CD31^+^CD144^+^ cells (76.0 ± 3.8%) (Figure 3b), which were similar to the proportions found in human umbilical vein endothelial cell populations (HUVECs) (Figure S1). As the proportion of CD31^+^CD144^+^ cells peaked at Passage 4, the molecular expression profile and functional performance of this population was assessed.

The expression of pluripotency markers, *OCT4* and *SOX2*, and mesoderm markers, *EOMES* and *MIXL1*, were downregulated 10- to 15-fold in the hiPSC-derived cells at Passage 4 compared with the starting hiPSCs (*p* = 0.0001–0.0002). In contrast, all endothelial cell markers *CD31*, *CD144*, *VEGFR2*, and *VWF* were significantly upregulated (*p* < 0.0001; Figure 3c). Because of the risk that pluripotent stem cell-derived epithelial cells can be misidentified as endothelial cells [34], an additional test for expression of *CDH1* and *EPCAM* was undertaken, which showed that these epithelial cell markers were strongly downregulated in hiPSC-ECs compared with an epithelial cell line control (*p* < 0.0001; Figure S2).

A key feature of endothelial cells is their ability to form tube-like structures. This functionality was probed with a time-course matrix tubulogenesis assay. The Passage 4 cells formed network structures within 3 h of plating atop Matrigel. These structures continued extending up to 6 h, after which the networks remodelled into circular cell clusters by 36 h (Figure 3d, Video S1).

### 3.2. hiPSC-Derived Endothelial Cells Form Viable and Aligned EngNT

**Figure 4:**
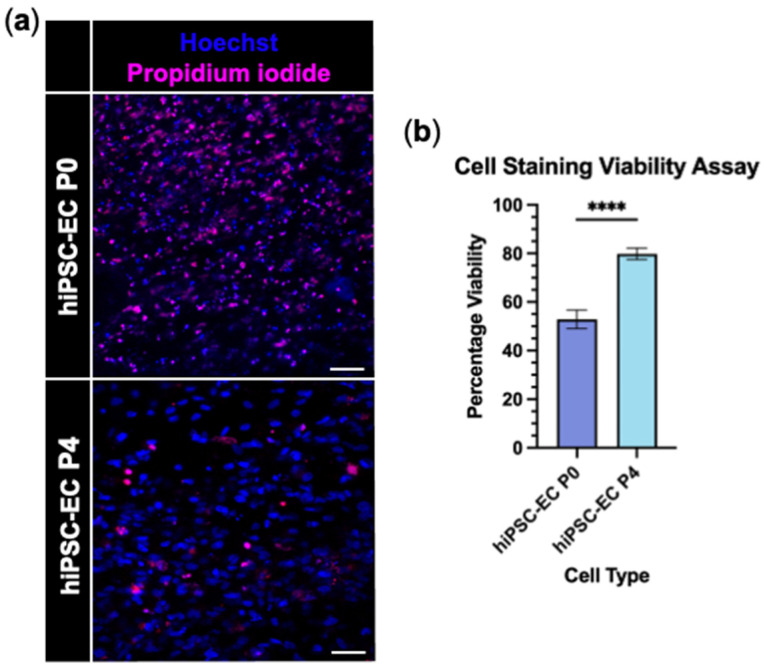
hiPSC-EC P4 are viable within EngNT. (**a**) Representative confocal micrographs of ReadyProbes™ stained EngNT containing hiPSC-EC P0 (top) or hiPSC-EC P4 (bottom). Dead cells stained with propidium iodide (magenta) and all cells stained with Hoechst 33342 (blue). Scale bar 50 µm. (**b**) Quantified percentage cell viability according to ReadyProbes™ staining. Data are mean ± SEM with unpaired *t*-test; **** *p* < 0.0001. Assay performed 24 h after GAE. N = 9 EngNT constructs.

Engineered neural tissue (EngNT) was fabricated via the gel aspiration-ejection technique, using hiPSC-derived endothelial cells from P0 and P4 populations. EngNT constructs containing hiPSC-EC P4 showed significantly higher viability than those made using hiPSC-EC P0, with cell viability of 79.7 ± 2.4% versus 52.9 ± 3.9% (*p* < 0.0001; Figure 4a,b).

**Figure 5:**
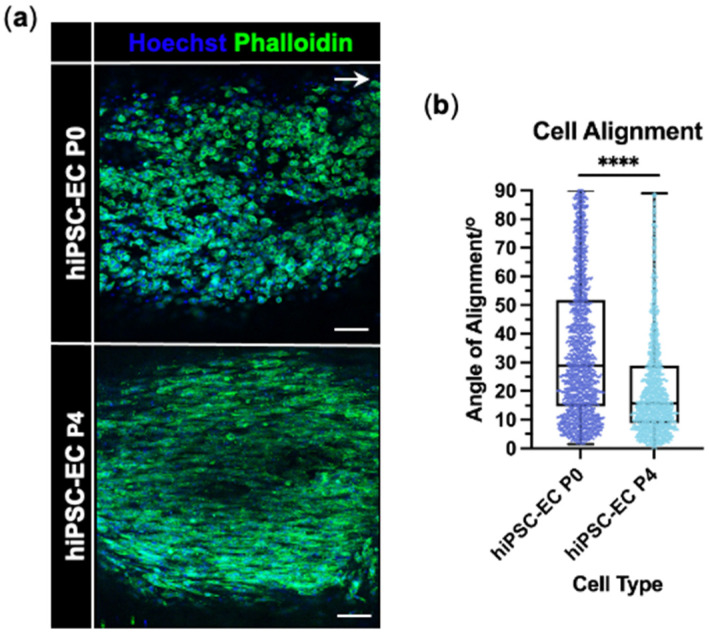
hiPSC-EC P4 are aligned and viable within EngNT. (**a**) Representative confocal micrographs of hiPSC-EC P0 (top) and hiPSC-EC P4 (bottom) within EngNT orientated along the longitudinal axis (white arrow). Cells stained with phalloidin (green) and Hoechst 33342 (blue). Scale bar 100 µm. (**b**) Angle of cell alignment quantified for hiPSC-EC P0 and hiPSC-EC P4 within EngNT with individual cells as data points; boxes show lower and upper quartile with median, whiskers show min and max, with Mann–Whitney test; **** *p* < 0.0001. Assay performed 24 h after GAE. N = 9 EngNT constructs.

The alignment of hiPSC-EC P4 parallel to the longitudinal axis within EngNT was significantly improved compared with hiPSC-EC P0 (Figure 5a,b). While both cell types showed alignment, the median cell alignment angle significantly decreased from 29.5° for hiPSC-EC P0 to 16.0° for hiPSC-EC P4 (*p* < 0.0001). The distribution of the cell angle of alignment for hiPSC-EC P4 also showed a greater positive skew than that of hiPSC-EC P0 (Figure 5b).

Based on the superior performance of hiPSC-EC P4 in EngNT viability and alignment, the hiPSC-ECs were used at P4 in all subsequent work.

### 3.3. hiPSC-EC Form Aligned Tube-like Structures Within EngNT

**Figure 6:**
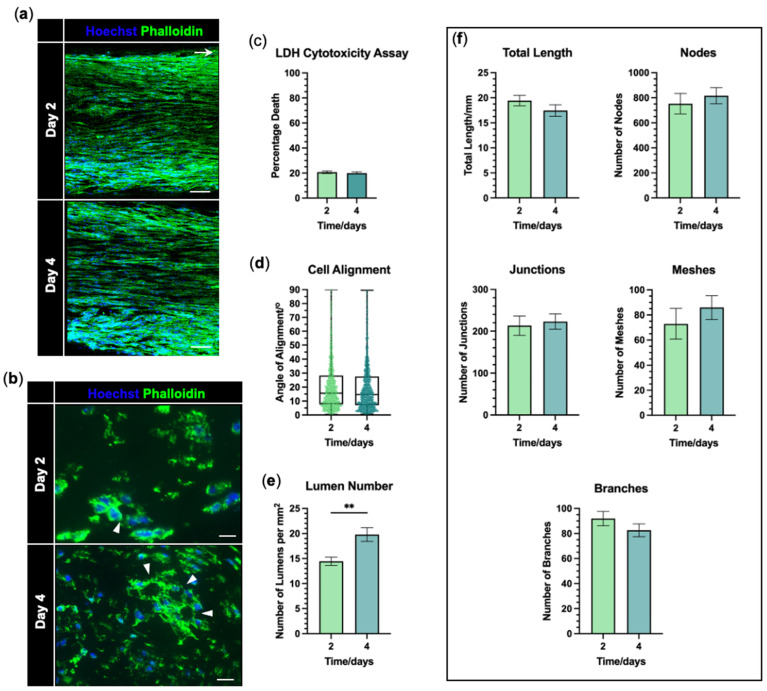
hiPSC-EC formed aligned tube-like structures within EngNT. (**a**) Representative confocal micrographs of hiPSC-ECs within EngNT-EC after 2 (top) and 4 (bottom) days in culture. Stained with phalloidin (green) and Hoechst 33342 (blue). Cellular structures orientated parallel to the longitudinal axis (white arrow). Scale bar 100 µm. (**b**) Transverse sections through EngNT-EC showing lumens as a ring of phalloidin fluorescence (green) with a black centre (white arrow heads). Scale bar 20 µm. (**c**) Percentage death of cells within EngNT-EC assessed by LDH cytotoxicity assay. (**d**) The angle by which the multinucleated structures deviate from the longitudinal axis, showing individual structures as data points. Boxes show lower and upper quartiles with median; whiskers show min and max. (**e**) Quantification of lumen number per mm^2^ from EngNT-EC cross-sections taken halfway along the length of the construct. (**f**) Total length of the multinucleated structures within EngNT-EC and angiogenic network measures of the multinucleated structures within EngNT-EC, quantified by ImageJ plugin Angiogenesis Analyzer. Data are mean ± SEM, unless otherwise stated, with unpaired *t*-test or Mann–Whitney; ** *p* < 0.01. N = 9 EngNT-EC constructs.

EngNT-EC constructs were maintained in culture and the formation of tube-like structures was assessed after 2 and 4 days. At each time point, the hiPSC-ECs formed numerous highly aligned multinucleated structures within EngNT-EC (Figure 6a) with lumens seen in the EngNT-EC cross-sections (Figure 6b). There was no increase in cell death over the 4 days in culture, with percentage death remaining around 20% (*p* = 0.3704; Figure 6c). The multinucleated cell structures were highly orientated parallel to the longitudinal axis of the construct, as shown by the low median alignment angle of these structures; 16.4° at 2 days and 14.8° at 4 days. There was no significant change in the alignment of the multinucleated cell structures over time (*p* = 0.8633; Figure 6d). The number of lumens per mm^2^ increased from 14.4 ± 0.8 to 19.8 ± 1.4 between 2 and 4 days in culture (*p* = 0.0041; Figure 6e). However, neither the total length of the highly aligned vascular-like structures nor any of the other angiogenesis measures exhibited any significant change over time (*p* = 0.2214–0.7425; Figure 6f).

### 3.4. EngNT-EC Constructs Support Neuronal Outgrowth In Vitro

**Figure 7:**
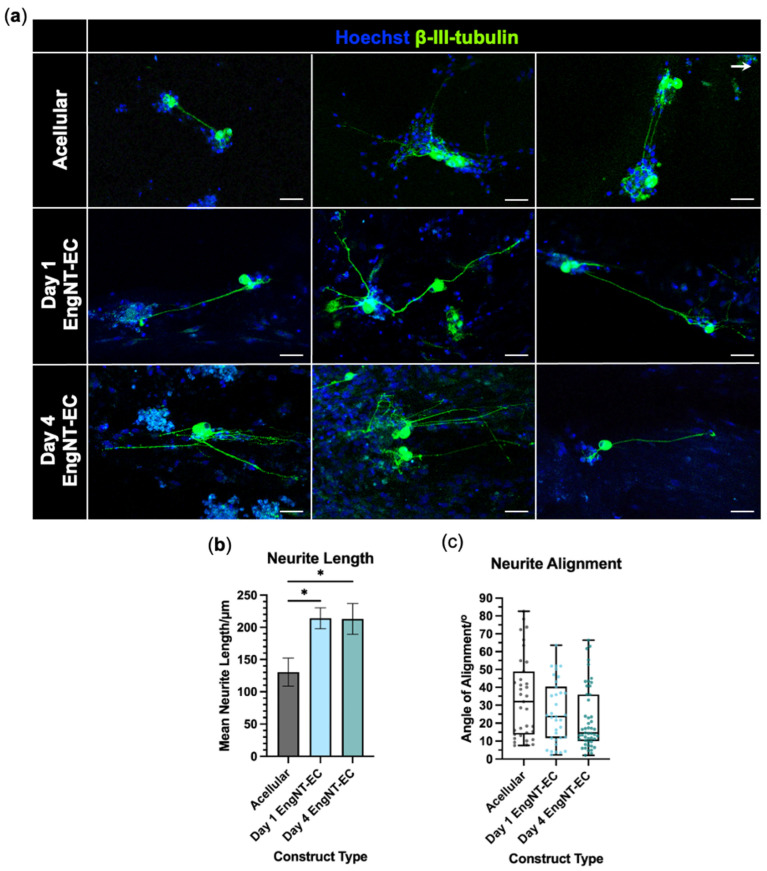
EngNT-EC constructs support and guide neurite growth in vitro. (**a**) Representative confocal micrographs of rat DRG neurons seeded onto the cut surface of longitudinally halved acellular constructs (top), EngNT-EC cultured for 1 day prior to co-culture (middle) and EngNT-EC cultured for 4 days prior to co-culture (bottom). Stained with anti-β-III tubulin (green) and Hoechst 33342 (blue). Arrow shows the direction of the longitudinal axis. Scale bar 50 µm. (**b**) Mean neurite length of neurons cultured on acellular constructs, and Day 1 and Day 4 EngNT-EC constructs. Data are mean ± SEM with Kruskal–Wallis test and Dunn’s comparison test; * *p* < 0.05. (**c**) Angle of neurite alignment on acellular constructs, and Day 1 and Day 4 EngNT-EC constructs. Boxes show lower and upper quartiles with median; whiskers show min and max, with individual neurites as data points. Kruskal–Wallis test showed no significant differences. N = 5 EngNT-EC constructs.

EngNT-EC constructs were longitudinally halved, exposing their inner environment, and cultured with rat DRG-derived neurons for 72 h. Acellular constructs were compared with EngNT-EC constructs that had been maintained in culture either for 1 day or for 4 days after fabrication, to allow any potential neuro-regenerative effects of tube-like structure maturation to be detected. All constructs were compatible with neurite extension, with neurites tending to grow parallel to the longitudinal axis (Figure 7a). Neurite length was significantly greater in EngNT-ECs compared with acellular constructs, increasing from ~130 µm in the acellular constructs to ~215 µm in both the Day 1 (*p* = 0.0193) and Day 4 (*p* = 0.0433) EngNT-EC constructs. There was no significant difference in neurite length between the Day 1 and Day 4 EngNT-EC constructs (*p* > 0.9999) (Figure 7b). The median neurite angle of alignment decreased from 35.1° for the acellular construct to 16.8° and 16.6° for Day 1 and Day 4 EngNT-EC, respectively (Figure 7c).

## 4. Discussion

The differentiation of human endothelial cells from hiPSCs offers a potential source of expandable endothelial cells which overcomes the variability of primary endothelial cell sources [35]. Here, endothelial cells were successfully differentiated from hiPSCs with the resulting cells expressing endothelial cell markers at the protein and mRNA level, in the absence of pluripotency and mesoderm markers, and demonstrating tubulogenesis functionality. The hiPSC-ECs were used to produce viable and aligned EngNT-EC constructs, fabricated using automated gel aspiration-ejection technology [28]. The endothelial cells formed tube-like structures within EngNT-EC constructs, which mimicked key features of the orientated vascular structures shown to be important at an early stage in nerve regeneration [10,11].

hiPSCs were differentiated to endothelial cells following a previously established protocol [25], a modification of the well-cited and rapid protocol developed by [33], which optimised differentiation efficiency, reduced the variability between iPSC sources, and replaced Matrigel with GMP-grade Geltrex during differentiation. Morphological changes observed here throughout differentiation mirrored those previously reported [25,33]. Hildebrant et al. reported ~80% differentiation efficiency according to the percentage of CD144^+^ cells on Day 6 [25]. Here, 33.0 ± 1.3% of cells were CD144^+^ on Day 6 of differentiation; this percentage increased to 92.6 ± 3.4% after culture in the presence of transforming growth factor-beta (TGF-β) inhibitor, SB431542, in microvascular endothelial cell-tailored medium, EGM™-2, for four passages. During the differentiation process, endothelial induction with the addition of VEGFA and forskolin occurred only for 2 days, between Day 4 and Day 6. This is a relatively short endothelial induction period compared with other published methods [36,37,38,39,40,41,42,43,44,45]. Hildebrandt et al., 2021, and others using modifications of Patsch et al., 2015, cultured the resulting cells for another 3–6 days before use [46,47,48], with Patsch et al., 2015, recommending that differentiated endothelial cells be used between Passages 3 and 4. This suggests that a subsequent maturation period may be beneficial for endothelial cell identity. Both the viability and alignment of hiPSC-ECs in EngNT-EC were greater when cells had been passaged to P4, confirming the suitability of the hiPSC-EC P4 cells for tissue engineering. The ~80% viability of hiPSC-EC P4 within EngNT corresponds to the viability of the rat Schwann cell line SCL4.1/F7 and HUVECs previously tested in EngNT by Muangsanit et al., [49] and Smith et al., [28]. It would be interesting in future studies to explore the suitability of cells from later passages for tissue engineering, since a stable endothelial cell phenotype was demonstrated from P4 to P10. Understanding the extent to which hiPSC-EC cultures can be expanded without loss of efficacy will be important for future translational development of the approach.

A defining characteristic of endothelial cells is their ability to form tubules. hiPSC-ECs at P4 formed network structures in the matrix tubulogenesis assay, confirming the presence of endothelial cell functionality [26]. The hiPSC-EC P4 formed network structures within 3 h, which remained stable up to 6 h, with the network disconnected by 24 h. This aligns with observations reported previously for the mouse lymphoid endothelial cell line, 3B-11 [26], and for HUVECs [50], with endothelial cells becoming apoptotic by 18–24 h, causing the network disintegration.

On culturing EngNT-EC for 2 or 4 days, cells formed an angiogenic network with a myriad of highly aligned tube-like structures. This was also seen with the previously reported EngNT containing HUVECs (EngNT-HUVEC) [28]. The highly directional and long angiogenic network present throughout the EngNT-EC mimics key features of the aligned vasculature formed within the regenerating nerve bridge [10]. The lack of significant changes between time points in the angiogenic measures of the network formed within EngNT-EC suggests that the network remains stable over at least 4 days in vitro. However, the significant increase in lumen number between 2 and 4 days in culture indicates a maturation of the network without network expansion [51]. Using endothelial cells to provide guidance rather than perfusable networks contrasts with other studies where endothelial cells have been used to pre-vascularise engineered tissue constructs [4,5,6]. As a result, other studies reported an additional endothelial cell network maturation time required for the vascular structures to be perfusable once implanted in vivo [52].

The presence of endothelial cells within EngNT constructs improved neurite growth and alignment to a similar extent, regardless of whether they were cultured for 1 or 4 days before seeding the neurons. The significant increase in neurite length in EngNT-ECs compared with acellular constructs could suggest that a positive interaction occurred between hiPSC-ECs and neurons. Whether this positive interaction was due to factors released from endothelial cells, such as BDNF and glial cell line-derived neurotrophic factor (GDNF) [8,53], and/or a direct physical interaction between the neurons and endothelial cells [10], would require further investigation. Future work could use these EngNT constructs as a model to explore the relative contributions of direct and indirect interactions between endothelial cells and neurons.

The neurite alignment conferred by the EngNT-EC constructs was comparable to that previously reported in EngNT containing differentiated adipose-derived stem cells [54] and EngNT containing SCL4.1/F7 Schwann cells [49]. The work of Muangsanit et al., 2020, also saw a similar improvement in neurite alignment in cellular EngNT versus acellular constructs [49].

This work developed the first stabilised engineered neural tissue construct containing columns of aligned endothelial cell tube-like structures which mimic key features of the polarised vasculature formed in the early nerve bridge, previously identified to be crucial for efficient nerve regeneration [10,11]. This provides a tool with which to probe the role of aligned endothelial tube-like structures in a peripheral nerve injury treatment. While the key endothelial cell differentiation markers were included in this study to monitor differentiation progression and characterise the resulting cells, the marker panel was limited and may not fully capture the complexity and heterogeneity of endothelial cell identity. Additionally, a limited number of time points were analysed for the endothelial cell tube-like structure formation within EngNT-ECs, which prevented the assessment of the long-term stability of these structures. Other limitations of this study include the species mismatch in the neurite outgrowth assay, which cultured rat sensory neurons on the EngNT-EC containing hiPSC-ECs. This was necessary because of the lack of availability of mature human neurons. In future studies, EngNT-EC constructs should be tested in vivo, for example, to repair a gap in a rat sciatic nerve. This could include a time course analysis to investigate the interaction of the construct with the host tissue, including integration with vasculature and perfusion of the endothelial tube-like structures, as well as the long-term stability of the structures. Moreover, this approach could be used to test whether the presence of endothelial cell tube-like structures in nerve repair constructs can improve indicators of regeneration such as axon growth and functional recovery.

## 5. Conclusions

hiPSCs were successfully differentiated into endothelial cells with the expression of pluripotency markers decreasing and endothelial cell markers increasing over the course of differentiation at the protein and mRNA level. The differentiated cells displayed endothelial cell functionality, forming network structures in the matrix tubulogenesis assay. These hiPSC-ECs were then applied to nerve tissue engineering to produce EngNT constructs containing endothelial cell tube-like structures capable of supporting neuronal outgrowth in vitro, and therefore providing a system which could be used to study the potential regenerative benefit of pre-formed aligned vascular-like structures in the treatment of traumatic peripheral nerve injury.

## Figures and Tables

**Table 1:** Antibody manufacturers and dilutions.

Antibody	Species	Manufacturer	Dilution	Catalogue
CD31 Polyclonal Antibody	Rabbit	Invitrogen, Paisley, UK	1:200	PA5-32321
CD144 (VE-cadherin) Monoclonal Antibody (16B1), eBioscience™	Mouse	Invitrogen, Paisley, UK	1:200	14-1449-82
Oct3/4 Antibody (C-20)	Goat	Santa Cruz, Welwyn Garden City, UK	1:200	SC-8629
Alexa Fluor^®^ 488 Anti-beta III tubulin antibody	Mouse	Abcam, Cambridge, UK	1:500	ab195879
Horse anti-goat IgG Antibody 594 DyLight^®^	Horse	VectorLabs, London, UK	1:200	DI-3094-1.5
Goat anti-mouse IgG Alexa Fluor™ Plus 488	Goat	Invitrogen, Paisley, UK	1:500	A21121
Goat anti-rabbit IgG Alexa Fluor™ Plus 488	Goat	Invitrogen, Paisley, UK	1:500	A32731
Goat anti-rabbit IgG Alexa Fluor™ Plus 647	Goat	Invitrogen, Paisley, UK	1:500	A32733

## Data Availability

All original experimental data are available at https://doi.org/10.5522/04/27194268.v2.

## Supplementary Materials

The following supporting information can be downloaded at https://www.mdpi.com/article/10.3390/jfb16110425/s1, Table S1: RT-qPCR primer sequences, Table S2: Statistical analysis of differences in the percentage of CD31^+^, CD144^+^, and CD31^+^CD144^+^ cells across hiPSC-EC passages; Figure S1: HUVEC immunostaining and tubulogenesis; Figure S2: Epithelial marker expression is significantly downregulated in hiPSC-ECs; Video S1: Matrix gel tubulogenesis assay of hiPSC-ECs at Passage 4.

## Abbreviations

The following abbreviations are used in this manuscript:

16G	16 gauge
Ara-C	Cytosine arabinoside
ATMP	Advanced therapy medicinal product
BDNF	Brain-derived neurotrophic factor
BMP4	Bone morphogenetic protein 4
cDNA	Complementary deoxyribose nucleic acid
D-PBS	Dulbecco’s phosphate-buffered saline
DMEM	Dulbecco’s Modified Eagle Medium
DMSO	Dimethyl sulfoxide
DRG	Dorsal root ganglion
EDTA	Ethylenediaminetetraacetic acid
EGM-2	Endothelial Growth Medium-2
EngNT	Engineered neural tissue
EngNT-EC	Engineered neural tissue containing endothelial cells
FBS	Foetal bovine serum
GAE	Gel aspiration-ejection
GDNF	Glial cell line-derived neurotrophic factor
GSK-3	Glycogen synthase kinase-3
hiPSCs	Human induced pluripotent stem cells
hiPSC-ECs	Human induced pluripotent stem cell-derived endothelial cells
HUVECs	Human umbilical vein endothelial cell
LDH	Lactate dehydrogenase
PBS	Phosphate-buffered saline
PCR	Polymerase chain reaction
PDL	Poly-D-lysine
Pen-Strep	Penicillin-streptomycin
PFA	Paraformaldehyde
ROCK	Rho-associated protein kinase
TGF-β	Transforming growth factor-β
VEGF(A)	Vascular endothelial growth factor (A)
