# Supplementary material for: Endothelial Cells Differentiated from Human Induced Pluripotent Stem Cells Form Aligned Network Structures in Engineered Neural Tissue
Source: J Funct Biomater. 2025 Nov 20;16(11):425. doi: 10.3390/jfb16110425 (PMC12653472; doi:10.3390/jfb16110425)
Supplement: Supplementary file 1 [file jfb-16-00425-s001.zip › POSmith JFB Supplementary.pdf]

Endothelial cells differentiated from human induced pluripotent stem cells form aligned network structures in engineered neural tissue

Poppy O. Smith [1,*], Parmjit Jat [2] and James B. Phillips [1]

[1]UCL Centre for Nerve Engineering, Department of Pharmacology, UCL School of Pharmacy, University College London, London, Greater London, WC1N 1AX, UK; poppy.smith.20@ucl.ac.uk (POS)
[2]MRC Prion Unit at UCL and Institute of Prion Diseases, University College London, London, Greater London, W1W 7FF, UK; p.jat@prion.ucl.ac.uk
*Correspondence: poppy.smith.20@ucl.ac.uk

Supplementary Material

Table S1. RT-qPCR primer sequences

Gene	Alias	Name	Forward Primer	Reverse Primer
CD31	EndoCAM, GPIIA′, PECA1, PECAM1,	Platelet and endothelial cell adhesion molecule 1	CCCAGCCCAGGATTTCTTATG	AGGCAAAGTTCCACTGATCG
CD144	7B4, CDH5, VE-Cadherin, VECD, VEcad, Vec	Cadherin 5	CCCTCACGGATAATCACGATAAC	GGAAGTGGACCTTGGTATGC
CDH1	Arc-1, BCDS1, CD324, CDHE, ECAD, LCAM, UVO	Cadherin 1	GCAAATTCCTGCCATTCTGG	CTCTTTGACCACCGCTCTC
CMYCERTAM	-	-	GAAAAGGCCCCCAAGGTAGT	TCTGGTCAGCTGTCAAGGAC
ENG	END, HHT1, ORW1	Endoglin	ATACCACTAGCCAGGTCTCG	GGACATGGACTTCAAGGATGG
EOMES	TBR2	Eomesodermin	GCTCTTCTTGGATAGAGACACC	AGCCGCCTTCGCTTAC
EPCAM	Ber-Ep4, BerEp4, DIAR5, EGP-2, EGP314, EGP40, ESA, HNPCC8, KS1/4, KSA, LYNCH8, M4S1, MIC18, MK-1, MOC-31, TACSTD1, TROP1	Epithelial cell adhesion molecule	GTGGTGATAGCAGTTGTTGC	GCCTTCTCATACTTTGCCATTC
HPRT1	HPRT, HGPRT	Hypoxanthine phosphoribosyl transferase 1	AGGGTGTTTATTCCTATGGAC	CCCATCTCCTTCATCACATCTC

MIXL1	MILD1, MIX, MIXL	Mix paired-like homeobox	TTCAGTTACCCTCCC AGATAAC	GGAGTGACTTTAA GACCAAAGC

Table S2. Statistical analysis of differences in the percentage of CD31+, CD144+ and CD31+CD144+ cells across hiPSC-EC passages.

**Comparison**	**CD31⁺**		**CD144⁺**		**CD31⁺CD144⁺**	
	**Summary**	**p Value**	**Summary**	**p Value**	**Summary**	**p Value**
P0 vs. P1	**	0.0012	****	<0.0001	ns	0.362
P0 vs. P2	ns	0.2104	****	<0.0001	ns	0.4384
P0 vs. P3	***	0.0002	****	<0.0001	ns	0.059
P0 vs. P4	ns	0.3112	****	<0.0001	****	<0.0001
P0 vs. P5	ns	>0.9999	****	<0.0001	****	<0.0001
P0 vs. P6	ns	0.9108	****	<0.0001	****	<0.0001
P0 vs. P7	ns	0.7576	ns	0.7291	**	0.0046
P0 vs. P8	ns	0.9919	****	<0.0001	****	<0.0001
P0 vs. P9	ns	0.9642	****	<0.0001	****	<0.0001
P0 vs. P10	*	0.0112	****	<0.0001	****	<0.0001
P1 vs. P2	ns	0.4138	****	<0.0001	**	0.003
P1 vs. P3	ns	0.9995	****	<0.0001	***	0.0002
P1 vs. P4	****	<0.0001	***	0.0009	****	<0.0001
P1 vs. P5	***	0.0008	ns	0.9944	****	<0.0001
P1 vs. P6	****	<0.0001	ns	>0.9999	****	<0.0001
P1 vs. P7	ns	0.0715	****	<0.0001	ns	0.55
P1 vs. P8	***	0.0001	ns	0.0547	****	<0.0001
P1 vs. P9	****	<0.0001	ns	0.1139	****	<0.0001
P1 vs. P10	****	<0.0001	ns	>0.9999	****	<0.0001
P2 vs. P3	ns	0.1233	ns	0.7963	ns	0.9823
P2 vs. P4	***	0.0008	****	<0.0001	****	<0.0001
P2 vs. P5	ns	0.148	****	<0.0001	****	<0.0001
P2 vs. P6	**	0.01	****	<0.0001	****	<0.0001
P2 vs. P7	ns	0.993	****	<0.0001	****	<0.0001
P2 vs. P8	*	0.0273	****	<0.0001	****	<0.0001
P2 vs. P9	*	0.0159	****	<0.0001	****	<0.0001
P2 vs. P10	****	<0.0001	****	<0.0001	****	<0.0001
P3 vs. P4	****	<0.0001	****	<0.0001	****	<0.0001
P3 vs. P5	***	0.0001	****	<0.0001	****	<0.0001
P3 vs. P6	****	<0.0001	****	<0.0001	****	<0.0001
P3 vs. P7	*	0.0148	****	<0.0001	****	<0.0001
P3 vs. P8	****	<0.0001	****	<0.0001	****	<0.0001
P3 vs. P9	****	<0.0001	****	<0.0001	****	<0.0001
P3 vs. P10	****	<0.0001	****	<0.0001	****	<0.0001
P4 vs. P5	ns	0.4142	**	0.0082	*	0.0129
P4 vs. P6	ns	0.9863	***	0.0005	ns	0.2797
P4 vs. P7	**	0.008	****	<0.0001	****	<0.0001
P4 vs. P8	ns	0.883	ns	0.743	**	0.0044
P4 vs. P9	ns	0.956	ns	0.5198	**	0.0052
P4 vs. P10	ns	0.8346	**	0.0023	ns	0.974

P5 vs. P6	ns	0.9621	ns	0.9749	ns	0.8898
P5 vs. P7	ns	0.6405	****	<0.0001	***	0.0003
P5 vs. P8	ns	0.9985	ns	0.3291	ns	>0.9999
P5 vs. P9	ns	0.9888	ns	0.5361	ns	>0.9999
P5 vs. P10	*	0.0174	ns	>0.9999	ns	0.1597
P6 vs. P7	ns	0.0873	****	<0.0001	****	<0.0001
P6 vs. P8	ns	>0.9999	*	0.0339	ns	0.6418
P6 vs. P9	ns	>0.9999	ns	0.0729	ns	0.687
P6 vs. P10	ns	0.23	ns	0.9998	ns	0.9232
P7 vs. P8	ns	0.2033	****	<0.0001	***	0.0009
P7 vs. P9	ns	0.1309	****	<0.0001	***	0.0007
P7 vs. P10	***	0.0002	****	<0.0001	****	<0.0001
P8 vs. P9	ns	>0.9999	ns	>0.9999	ns	>0.9999
P8 vs. P10	ns	0.1006	ns	0.1271	ns	0.0636
P9 vs. P10	ns	0.1594	ns	0.2438	ns	0.074

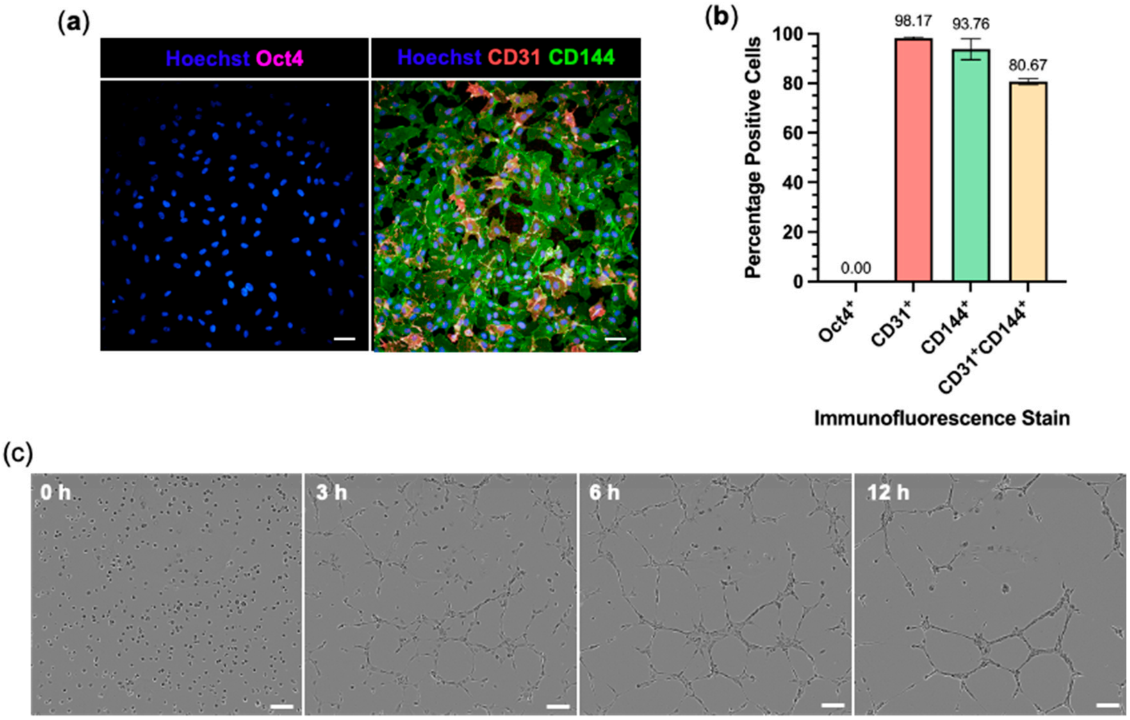

Figure S1. HUVEC immunostaining and tubulogenesis. (**a**) Representative immunofluorescence images show the proportion of HUVECs positive for pluripotency marker Oct4 (magenta) and endothelial cell markers CD31 (red) and CD144 (green). All cells stained with Hoechst 33342 (blue). Scale bar 50 µm. (**b**) Columbus Image Analysis Software quantification of immunofluorescence by percentage positive HUVECs for Oct4 (purple), CD31 (red), CD144 (green), and CD31 and CD144-double positive (yellow). Data mean ± SEM. (**c**) Representative phase contrast images of HUVECs at given times during the matrix gel tubulogenesis assay. The imaging frame was consistent over time. N = 3 independent passages with n = 3 technical replicates.

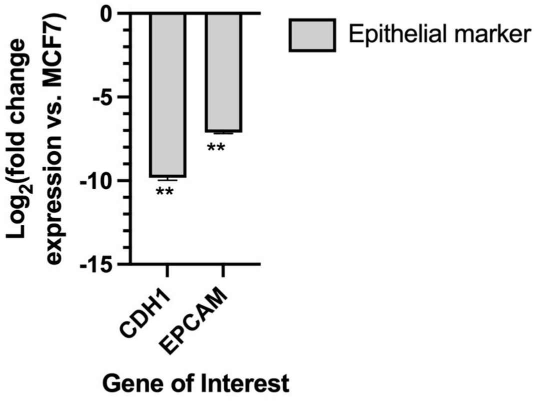

Figure S2. Epithelial marker expression is significantly downregulated in hiPSC-ECs. Log2-fold change mRNA expression of epithelial cell markers, CDH1 and EPCAM, in hiPSC-ECs P4 relative to the breast cancer cell line, MCF7, as the epithelial cell control. Data mean ± SEM with one-way ANOVA with Tukey's or Dunnett's comparison test; **$p < 0.01$. N = 3 independent differentiation inductions with n = 3 technical replicates.
